# Investigation of perioperative safety and clinical results of one-stage bilateral total knee arthroplasty in selected low-risk patients

**DOI:** 10.1186/s13018-018-0720-6

**Published:** 2018-01-17

**Authors:** Hirotaka Mutsuzaki, Arata Watanabe, Tetsuya Komatsuzaki, Tomonori Kinugasa, Kotaro Ikeda

**Affiliations:** 1 Department of Orthopaedic Surgery, Ibaraki Prefectural University of Health Sciences, 4669-2 Ami, Ami-machi, Inashiki-gun, Ibaraki 300-0394 Japan; 2 Department of Orthopaedic Surgery, Ichihara Hospital, 3681 Ozone, Tsukuba, Ibaraki 300-3295 Japan; 3 Department of Anesthesiology, Ichihara Hospital, 3681 Ozone, Tsukuba, Ibaraki 300-3295 Japan

**Keywords:** One-stage bilateral total knee arthroplasty, Complications, Clinical results, Blood loss, Safety

## Abstract

**Background:**

An increased perioperative complication rate has been a concern with one-stage bilateral total knee arthroplasty (TKA). The purpose of this study was to retrospectively investigate the perioperative safety and clinical results of one-stage bilateral TKA in selected low-risk patients.

**Methods:**

Sixty-seven patients who received one-stage bilateral TKAs for osteoarthritis who were American Society of Anesthesiology (ASA) class 1 or 2 were included in this study. Perioperative complications, blood loss, transfusion rate, blood laboratory results, and clinical results were evaluated up to 1 year after surgery.

**Results:**

No major complications (deep infection, pulmonary embolism, cerebrovascular accident, myocardial infarction, death, or removal or revision of the implants) were observed. The average total blood loss was 1139.5 ml. The transfusion rate was 95.5%. Postoperative hemoglobin level and C-reactive protein level gradually improved up to postoperative day 21 (*P* < 0.01). Bilateral knee extension knee angles and clinical scores improved postoperatively as compared with preoperative values (*P* < 0.01).

**Conclusions:**

Although total blood loss and transfusion rate can be high, this preliminary case series suggested that the one-stage bilateral TKA in ASA class 1 or 2 patients can have high perioperative safety levels, and good clinical results can be obtained up to 1 year after surgery. If low-risk patients are selected for bilateral TKA, a one-stage procedure can be beneficial for patients, with a minimal increase in the risk of complications.

## Background

Total knee arthroplasty (TKA) leads to good clinical results with excellent long-term survivorship in patients with osteoarthritis (OA) [1, 2]. One third of patients receiving TKA were found to have degenerative OA in both knees, and 20% of patients required a contralateral TKA within 2 years of undergoing their first surgery [3–5]. Bilateral TKA has two possible surgical strategies: a one-stage surgical procedure with a single session of anesthesia and one hospitalization, or two separate surgical procedures between 1 and 12 months apart. Proponents of the one-stage bilateral TKA cite the potential advantages of shorter overall recovery time, less time off work, a single administration of anesthesia, and decreased total cost when compared with a two-stage TKA [6–8]. However, the risk of perioperative complications associated with one-stage bilateral TKA has been shown to be slightly increased compared with unilateral or two-stage bilateral TKA [9–11]. In contrast, others have reported that one-stage and two-stage bilateral TKA had a similar incidence of postoperative complications [12, 13]. However, these patients were not selected using a preoperative risk assessment prior to surgery.

In American Society of Anesthesiology (ASA) class 1 or 2 patients with few or no associated comorbidities, a one-stage bilateral TKA procedure did not result in severe complications [14, 15]. To our knowledge, there have been no reports on the perioperative complications, blood loss, transfusion rate, blood laboratory results, and clinical results up to 1 year after one-stage bilateral TKA in ASA class 1 or 2 patients. The purpose of this study was to retrospectively investigate the perioperative safety and clinical results of one-stage bilateral TKA in selected low-risk patients.

## Methods

The ethics committee of Ichihara Hospital reviewed and approved this retrospective study. From January 2012 to December 2015, 67 patients underwent one-stage bilateral TKA for OA with a single session of anesthesia and one hospitalization.

Before surgery, a systematic outpatient anesthesia consultation took place with a standardized work-up including a chest X-ray and blood, urine, and cardiac examinations. Patients classified as ASA 1 or 2 received one-stage bilateral TKA. The age, sex, height, body weight, body mass index (BMI), and follow-up period are summarized in Table 1. Patients were hospitalized 1 day before surgery.

**Table 1:** Patient profiles

Parameter	*n* = 67
Age (years)	71.1 ± 6.1
Sex (male/female)	18/49
Disease	OA: 67
Height (cm)	152.2 ± 8.6
Body weight (kg)	62.4 ± 9.7
BMI (kg/m^2^)	29.6 ± 3.6
Follow-up period (months)	12.7 ± 4.2

*OA* osteoarthritis, *BMI* body mass index

Results are presented as mean ± standard deviation

The surgical procedure was similar to our previous report [16] and is described briefly below. General anesthesia with a femoral nerve block or spinal anesthesia was used for patients, and a tourniquet was inflated from incision to skin closure. Two separate surgeons worked simultaneously. After a midline skin incision was made, a medial parapatellar approach was used. An intramedullary alignment rod was used for the femoral cuts and an extramedullary or intramedullary guide system was used for the tibial cuts. The patella was not replaced if the articular cartilage remained. We used cement when we used implants that recommended cement fixation or when osteoporotic bone was present. NexGen CR-type (134 knees) (Zimmer, Warsaw, IN, USA) femoral components and the NexGen Stem (2 knees) or the NexGen Trabecular Metal Monoblock (132 knees) (all Zimmer components) tibial components were used.

The procedures for bleeding prevention, postoperative antibiotic therapy, and thromboprophylaxis prevention were similar to our previous report [16]. Autologous blood transfusions and allogeneic blood transfusions were also performed using the same protocol as previously described [16–18]. These levels were adjusted individually based on the cardiovascular status of patients. As part of the postoperative care for both groups, continuous passive movement began on postoperative day (POD) 3, and standing and full weight-bearing walking was allowed 1 week after surgery.

The number of major and minor complications after surgery was monitored. Major complications included deep infection, pulmonary embolism, cerebrovascular accident, myocardial infarction, death, or removal or revision of the implants. All other complications were considered minor.

Knee range of motion (ROM) was assessed preoperatively, at admission, and 1 year postoperatively, and the Japanese Orthopaedic Association OA knee rating score (JOA score) was evaluated, with a total maximum score of 100 points [19] preoperatively and 1 year after surgery. There were strong correlations between JOA score and Knee Society function score [20, 21]. Operative time and time to discharge after surgery were also monitored.

Hemoglobin levels were measured preoperatively and on PODs 1, 7, 14, and 21, along C-reactive protein (CRP) level on PODs 7, 14, and 21, and D-dimer level 7 days after surgery. Blood loss from drainage, total postoperative blood loss, and the need for autogenic and/or allogeneic blood transfusions were recorded. A formula proposed by Nadler et al. [22] and Sehat et al. [23] was used to calculate the total postoperative blood loss.

### Statistical analyses

For comparison between two time points, a paired *t* test was used. For comparison between more than three time points, a repeated one-way analysis of variance along with a Bonferroni/Dunn post hoc test were used. A *P* value ≤0.05 was considered a significant difference, and all analyses were performed using IBM SPSS Statistics (Version 24; SPSS, Chicago, IL, USA).

## Results

The results are summarized in Tables 2 and 3.

**Table 2:** Knee data

Parameter	*n* = 67
Major complication rate (%)	0
Minor complication rate (%)	14.9
Operative time (minutes)	89.6 ± 12.1
Time to discharge from operation (days)	43.8 ± 14.2
Extension angle on the right side (°)	
Preoperative	−10.9 ± 8.0^a^
At discharge	−2.8 ± 3.9^a^
At 1 year	−1.1 ± 2.4^a^
Extension angle on the left side (°)	
Preoperative	−11.6 ± 8.1^a^
At discharge	−2.3 ± 3.2^a^
At 1 year	−1.2 ± 2.5^a^
Flexion angle on the right side (°)	
Preoperative	117.5 ± 16.6
At discharge	109.3 ± 10.3^a^
At 1 year	113.2 ± 15.4
Flexion angle on the left side (°)	
Preoperative	118.6 ± 15.7
At discharge	108.7 ± 11.3^a^
At 1 year	114.8 ± 12.6
JOA score on the right side (points)	
Preoperative	53.4 ± 9.0^a^
At 1 year	86.9 ± 7.1^a^
JOA score on the left side (points)	
Preoperative	53.7 ± 9.0^a^
At 1 year	86.4 ± 7.9^a^

*ROM* range of motion, *JOA* Japanese Orthopedic Association

Results are presented as mean ± standard deviation

^a^Significant difference between the time points (*P* < 0.05)

**Table 3:** Blood examination, blood loss, and blood transfusion data

Parameter	*n* = 67
Hb (g/dl)	
Preoperative	12.8 ± 1.1^a^
POD 1	11.0 ± 1.1^a^
POD 7	10.2 ± 1.3^a^
POD 14	10.6 ± 1.1^a^
POD 21	10.8 ± 1.1 (*n* = 66)^a^
Postoperative CRP (mg/dl)	
POD 7	3.4 ± 1.7^a^
POD 14	1.7 ± 1.5^a^
POD 21	1.0 ± 1.2 (*n* = 66)^a^
D-dimer level 7 days after surgery (μg/ml)	13.4 ± 6.1 (*n* = 65)
Blood loss (ml)	
Drained	617.3 ± 362.6
Total	1139.5 ± 365.7
Transfusions	
Transfusion rate (%)	95.5
Autologous (ml)	310.4 ± 168.0
Allogeneic (units)	0.4 ± 0.8
Amount of Hb (g)	51.7 ± 10.2

*Hb* hemoglobin, *POD* postoperative day, *CRP* C-reactive protein

Results are presented as mean ± standard deviation

^a^Significant difference between the time points (*P* < 0.05)

No major complications (deep infection, pulmonary embolism, cerebrovascular accident, myocardial infarction, death, or removal or revision of the implants) were observed. The minor complications included three cases of wound dehiscence, two episodes of anemia, two hematomas, one urinary tract infection, one fever more than 10 day after surgery, and one case of delirium. The minor complication rate was 14.9%, but all of these complications were improved by the time of discharge. The bilateral knee extension angles gradually increased from before surgery, to discharge, to 1 year after surgery (*P* < 0.05). Although the knee flexion angle at discharge was smaller in both knees than that before surgery (*P* < 0.05), it had recovered a preoperative level at 1 year after surgery (*P* > 0.05). In the both knees, the 1-year postoperative JOA scores were higher than those before surgery (*P* < 0.01). The average operative time was 89.6 ± 12.1 min. The average time to discharge after operation was 43.8 ± 14.2 days, and all patients walked with or without a cane at the time of discharge.

The average total blood loss was 1139.5 ± 365.7 ml. The total transfusion rate (autologous plus allogeneic) was 95.5%. The average volume of allogeneic transfusion required was 310.4 ± 168.0 ml, and the average amount of autogenic transfusion was 0.4 ± 0.8 units. The hemoglobin level up to POD 21 was less than before surgery (*P* < 0.01). Hemoglobin level on POD 7 was the lowest (*P* < 0.01), and it gradually increased up to POD 21 (*P* < 0.01). The D-dimer level 7 days after surgery was 13.4 ± 6.1 μg/ml. The CRP level gradually decreased up to POD 21 (*P* < 0.01).

## Discussion

The most important finding of the present study was that a one-stage bilateral TKA in ASA class 1 or 2 patients who underwent screening by an anesthesiologist before the operation can be achieved with adequate perioperative safety and good clinical results up to 1 year after surgery.

No major complications such as deep vein thrombosis or pulmonary embolism occurred. An increased rate of deep vein thrombosis, pulmonary embolism, cardiac complications, and death in bilateral TKA has been reported [15, 24]. In particular, elderly patients and ASA class 3 or 4 patients have a major risk of severe complications [15]. However, in selected ASA class 1 or 2 patients, no perioperative death or pulmonary embolism was observed in one-stage bilateral TKA [25]. In this study, we also performed one-stage bilateral TKA in selected patients who were ASA class 1 or 2, and no major complications occurred. However, patients in the one-stage bilateral TKA in the present study had a greater postoperative blood loss, transfusion volume, and transfusion rate than in our previous report that used a similar procedure for unilateral TKA [16]. The hemoglobin level up to 3 weeks after surgery was less than before surgery. Patients require adequate preoperative preparation of blood and must be closely observed for heart rate and blood pressure changes. Moreover, the D-dimer level 7 days after surgery in this study was higher than that in our previous report that used a similar procedure for unilateral TKA [16]. Furthermore, the operative time in this study was longer than that in our previous report that used a similar procedure for unilateral TKA [16]. The one-stage bilateral TKA may lead to an increased risk of serious cardiac and pulmonary complications and mortality [26, 27]. Thus, the physiological insult of one-stage bilateral TKA can be larger than in unilateral TKA. Therefore, it is necessary to be cautious of major complications if one-stage bilateral TKA is selected as the method of treatment. We carefully selected the patients in which to safely perform the one-stage bilateral TKA using a systematic anesthesia consultation.

In our study, the knee extension angles in both knees gradually increase from before surgery, to discharge, to 1 year after surgery. The 1-year postoperative JOA score was higher than the score before surgery. The CRP level gradually decreased up to 3 weeks after surgery. Therefore, the postoperative course, including the perioperative course and clinical results, was considered good. The one-stage bilateral TKA is advantageous in having one hospitalization, a single anesthesia administration, and good postoperative clinical results. Some authors have reported that one-stage bilateral TKA has the advantage of decreasing the hospitalization period, having a shorter overall recovery time and reducing the financial burden to patients [6–8]. Since a one-stage bilateral TKA can be performed safely in low-risk patients, a one-stage procedure can be beneficial in low-risk patients.

Our study had limitations. Our sample size was small and our study was retrospective. Prospective randomized controlled studies with a larger number of patients are needed to clarify the safety and effectiveness of one-stage bilateral TKA in selected patients. Our follow-up period was relatively short because we were interested in perioperative safety and effectiveness. It may be necessary to study and include late complications.

## Conclusions

Although total blood loss, transfusion rate, and the physiological insult can be large, this preliminary case series suggested that a one-stage bilateral TKA in ASA class 1 or 2 patients can achieve favorable perioperative safety and good clinical results up to 1 year after surgery. If low-risk patients are selected for bilateral TKA, a one-stage procedure can be beneficial for patients, with a minimal increase in the risk of complications.

## Abbreviations

ASA: American Society of Anesthesiology
BMI: Body mass index
CRP: C-reactive protein
JOA score: Japanese Orthopaedic Association OA knee rating score
OA: Osteoarthritis
POD: Postoperative day
ROM: Range of motion
TKA: Total knee arthroplasty
